# The underestimated role of the microphthalmia-associated transcription factor (MiTF) in normal and pathological haematopoiesis

**DOI:** 10.1186/s13578-021-00529-0

**Published:** 2021-01-13

**Authors:** Alessia Oppezzo, Filippo Rosselli

**Affiliations:** 1 CNRS UMR9019, Équipe labellisée La Ligue contre le Cancer, Gustave Roussy, 114 rue Edouard Vaillant, 94805 Villejuif, France; 2 Gustave Roussy Cancer Center, 94805 Villejuif, France; 3 Université Paris Saclay - Paris Sud, Orsay, France

## Abstract

Haematopoiesis, the process by which a restrained population of stem cells terminally differentiates into specific types of blood cells, depends on the tightly regulated temporospatial activity of several transcription factors (TFs). The deregulation of their activity or expression is a main cause of pathological haematopoiesis, leading to bone marrow failure (BMF), anaemia and leukaemia. TFs can be induced and/or activated by different stimuli, to which they respond by regulating the expression of genes and gene networks. Most TFs are highly pleiotropic; i.e., they are capable of influencing two or more apparently unrelated phenotypic traits, and the action of a single TF in a specific setting often depends on its interaction with other TFs and signalling pathway components. The microphthalmia-associated TF (MiTF) is a prototype TF in multiple situations. MiTF has been described extensively as a key regulator of melanocyte and melanoma development because it acts mainly as an oncogene. *Mitf*-mutated mice show a plethora of pleiotropic phenotypes, such as microphthalmia, deafness, abnormal pigmentation, retinal degeneration, reduced mast cell numbers and osteopetrosis, revealing a greater requirement for MiTF activity in cells and tissue. A growing amount of evidence has led to the delineation of key roles for MiTF in haematopoiesis and/or in cells of haematopoietic origin, including haematopoietic stem cells, mast cells, NK cells, basophiles, B cells and osteoclasts. This review summarizes several roles of MiTF in cells of the haematopoietic system and how MiTFs can impact BM development.

## MiTF expression and activity

**Fig. 1:**
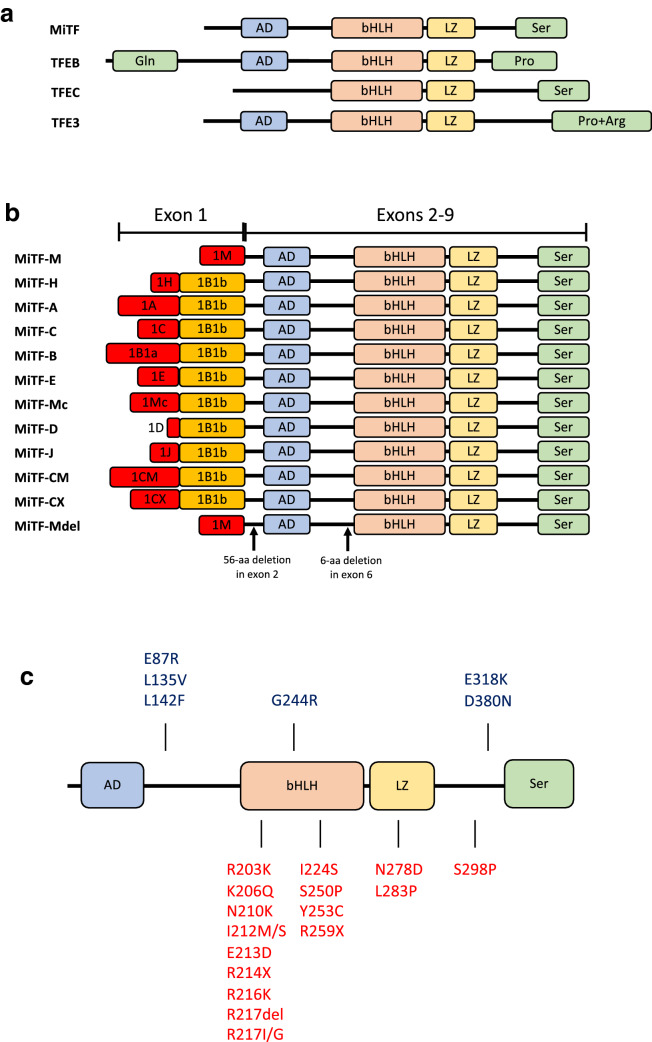
**a** Structure of the four MiT family members. AD, acidic domain; bHLH, basic helix-loop-helix; LZ, leucine zipper; Ser, serine-rich region; Gln, glutamine-rich region; Pro, proline-rich segment; Pro + Arg, proline- and arginine-rich region (adapted from [1^112^, ^186^]. **b** Different MiTF isoforms. Each isoform is driven by its own promoter and has a partially unique exon, while exons from 2 through 9 are common in all isoforms. **c** MITF mutations found in melanoma (blue, on top) and in pigment deficiency syndromes (WS2A and TS patients, red, bottom) (adapted from [37]

In 1942, Paula Hertwig, considering the progeny of irradiated mice, described animals that shared several pathological defects, including pigmentation loss, microphthalmia, deafness, osteopetrosis and a reduced number of mast cells (MCs) [45] and called them *mi/mi* (for microphthalmia) mice. The gene encoding the protein whose loss-of-function is critical for the *mi/mi* phenotype was cloned in 1993 and found to encode a transcription factor (TF) that was called *MiTF* for Microphthalmia Transcription Factor. MiTF is a member of the basic-helix-loop-helix-leucine zipper (bHLH-ZIP) family [48] characterized by three regions: an HLH and a ZIP motif, which are both involved in protein dimerization and required for the DNA binding mediated by a basic domain, which constitutes the third region [100] (Fig. 1).

MiTF forms both homodimers and heterodimers with three other bHLH-ZIP TFs (TFEB, TFEC and TFE3), all members of the MiT family (Fig. 1a), that regulate gene expression by binding to E-box motifs characterized by the core hexanucleotide sequence CA[C/T]GTG [42] or by establishing bridges with and modulating the activity of other TFs. MiTF and TFEC are expressed in a cell-restricted manner [188], while TFEB and TFE3 are ubiquitously expressed [8, 17].

As consequence of different transcription starting sites or alternative splicing of its full-length RNA, MiTF is produced in several isoforms (Fig. 1b). Two isoforms based on alternative promoter usage, the melanocyte-specific (*MiTF-M*) and heart-specific (*MiTF-H*) transcripts, were identified in 1994 (Steingrimsson et al. [161]). Other isoforms have been successively identified in different tissues and settings: MiTF-A [6, 185], MiTF-C [31]; MiTF-B [174], MiTF-E [117], MiTF-Mc [168], MiTF-D [167]; MiTF-J [44], and MITF-CM [154]. Finally, MiTF-CX was found to be highly expressed in the cervix during pregnancy [83] and MITF-Mdel, a splice variant of MITF-M, was identified in melanocytes and melanoma cell lines [179].

The existence of multiple isoforms of MiTF, characterized by complex patterns of tissue-specific expression and the propensity to dimerize with other partners, may help explain its pleiotropy and its different biological effects on various cell types.

MiTF expression and/or activity are regulated at both mRNA and protein levels. The major positive regulators of MiTF transcription comprise the SWI/SNF chromatin remodelling complex [175], the WNT/β-catenin pathway (Bellei et al. [10]), SOX10 (sex-determining region Y-box10) [52], and CREB (cAMP response element-binding protein) [143], which can be activated by different signalling pathways. Moreover, MiTF fosters the expression of its own gene by recruiting LEF-1/β-catenin to its promoter [144]. GLI2 (glioma-associated oncogene family member 2), a TF activated by TGFβ [129], DEC1 (differentially expressed in chondrocytes protein 1, which is recruited to the *MiTF* promoter by HIF1α (hypoxia-inducible factor 1α) [28], and c-MYC [132] are considered negative regulators of *MiTF* expression. Activated in response to TNF-α exposure, NF-kB can both induce and repress *MiTF* [64, 71]. Finally, *MiTF* transcripts can be either stabilized by their association with CRD-BP (coding region determinant-binding protein) (Craig and Spiegelman [21]) or degraded by their interaction with several miRNAs [9].

The transcriptional activity of MiTF is largely regulated post-translationally. Notably, MiTF is phosphorylated by ERK1/2 at Ser73, by p90 ribosomal S6 kinase (p90RSK) at Ser409, by glycogen synthase kinase-3β (GSK3β) at Ser298 and by p38 MAPK at Ser307 [40, 82, 182]. MiTF phosphorylation generally enhances its activity, even though double phosphorylation at Ser73 and Ser409 promotes its proteasome-dependent degradation [182]. SAEI/SAEII- and UBC9-mediated SUMOylation at Lys182 and Lys316 inhibit MiTF [110], which can also lead to its degradation by the proteasome following UBC9-mediated ubiquitylation of Lys201 [183] or cleaved by caspase-3 during the apoptotic process [75].

MITF activity also depends on the availability of cooperating partners that can serve as activators, such as p300/CBP [182], or repressors, such as the member of the histidine triad (HIT) protein family HINT1 (histidine triad nucleotide-binding protein 1) [33].

### Diseases associated with MiTF

MiTF is a recognized key TF in melanocyte biology and plays a fundamental role in melanoma, acting essentially as an oncogene. Indeed, in melanoma MiTF is generally overexpressed, with gene amplification observed in approximately 30% of the samples, or overactivated by phosphorylation in a B-RAF^V600E^/ERK-dependent manner [22, 64, 68].

In humans, germinal *MiTF* mutations have been mainly identified in the heterozygous state [137], and they are most frequently loss-of-function mutations that result in haploinsufficiency leading to pathologies associated with pigment abnormalities and congenital hearing loss, including Waardenburg syndrome type II (WS2) and Tietz albinism-deafness syndrome (TADS) [170, 177]. *MiTF* mutations cause type 2A WS2 (WS2A), accounting for 20% of all WS2 cases [137, 130]. Most identified mutations alter exons 7 and 8, encoding the b-HLH-Zip motifs involved in protein dimerization [130]. It has been suggested that *MiTF* mutations resulting in a truncated protein or in a protein unable to dimerize lead to WS2 through haploinsufficiency, whereas mutations exerting a dominant negative effect result in TADS, mostly characterized by non-truncating mutations in the basic domain [59, 156].

Supporting the possibility that, in contrast to mice, the activity of MiTF is required for normal human development, individuals bearing two inactivated alleles have rarely been identified. A homozygous intronic mutation of the 5′ splice site sequence affecting only the lineage-specific MiTF-M isoform has been associated with a more severe WS2A phenotype [135]. Another homozygous mutation (p.R223H) leads to a classic WS with persistent chronic constipation after the neonatal period, a symptom suggestive of Waardenburg syndrome type 4 (WS4), also known as Waardenburg-Shah syndrome [123, 124]. Finally, compound-heterozygous *MiTF* mutations have been identified in the severe multisystemic disorder termed COMMAD (coloboma, osteopetrosis, microphthalmia, macrocephaly, albinism, and deafness). These mutations are located in common exons (exons 2–9) and alter all MiTF isoforms, explaining the failure of multiple organ systems, and act in a dominant-negative manner, modifying nuclear localization and DNA-binding proficiency of MiTF homo- and heterodimers [34].

Interestingly, *MiTF* mutations associated with pigment deficiency syndromes and melanoma have different effects on protein function: mutations associated with WS2A and TADS are located in the bHLH-ZIP domains and prevent MiTF from binding DNA, while mutations found in melanoma cases are mostly located at the amino- or carboxy-termini affecting the transactivation potential of MiTF (Fig. 1c) [37].

### MiTF mouse models

Two KO mouse models have been specifically used to study MiTF biology. The first is the *mi/mi* model (see above). The *mi* mutant allele (*mi-Mitf*) encodes a protein that has lost 1 of 4 consecutive arginine residues in the basic domain, which makes mi-Mitf defective in both DNA binding and nuclear localization (Steingrimsson et al. [161]; Morii et al. [101]. The other is the *tg/tg* mouse model, which does not express any Mitf isoform due to an insertional mutation that destroys the promoter region of *Mitf* [166]. Notably, the phenotypic abnormalities of the *tg/tg* mice are relatively mild compared to those observed in the *mi/mi* mice. Indeed, the transcription of several genes is more profoundly affected in *mi/mi* than it is in *tg/tg* cells, indicating that, in addition to the loss of its transactivation ability, mi-Mitf exerts transcription dominant-negative effects sequestering partners outside the nucleus [54, 62].

### MiTF and haematopoiesis

**Fig. 2:**
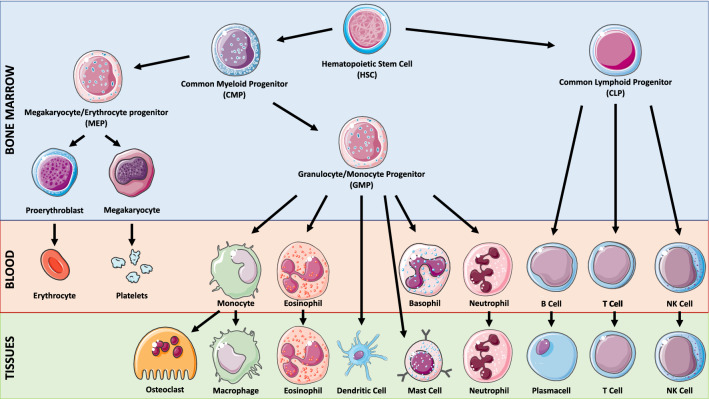
Simplified representation of the haematopoietic hierarchy. Haematopoiesis has a hierarchical organization with HSCs on top and mature blood cells at the bottom. HSCs are the sources of various types of progenitor cells that proliferate extensively, forming more differentiated cells at the expense of their self-renewal capacity

The haematopoietic system is a pyramidal organization with multipotent haematopoietic stem cells (HSCs) at the apex and mature blood cells at the bottom, with haematopoiesis being the process by which the naive population of HSCs are terminally differentiated into various blood cells. HSCs are defined by dormancy, the ability to remain out of the cell cycle for long periods (up to years), self-renewal, the ability to form stem cells, and multipotency, the ability to generate progenitor intermediates, which in turn will differentiate into several blood cell lineages [24, 122]. HSCs develop into multipotent progenitors (MPPs) that differentiate into common lymphoid progenitors (CLPs), precursors of all lymphoid cells, or common myeloid progenitors (CMPs), precursors of all myeloid cells [24, 138] (Fig. 2).

Normal haematopoiesis depends on the tightly regulated temporospatial activity of several TFs. The deregulation of their activity or expression is a main cause of pathological haematopoiesis, leading to bone marrow failure (BMF), anaemia and leukaemia. Although the role of MiTF in haematopoiesis is still unclear in humans, the analysis of the *mi/mi* and *tg/tg mice* enabled us to highlight MiTF importance in the haematopoietic context, mainly in the differentiation of some haematopoietic cell lineages: B lymphocytes, natural killer (NK) cells, mast cells (MCs), and osteoclasts (OCs).

B and NK cells develop from CLPs. B lymphocytes constitute a major component of humoral immunity. As pre-B cells, they differentiate in the BM, where they undergo the process of V(D)J recombination that leads to their immunoglobulin heavy and light chain formation, which is the basis of the B cell receptor (BCR). These immature B cells mainly migrate into the spleen, where they become activated B cells when their BCR binds its cognate antigen. Activated B cells undergo somatic hypermutation (SHM) and class switch recombination (CSR) process, which increases the affinity of the antibodies for their antigens and determines the class of specific class of B cells, respectively. Finally, the B cells become either antibody-secreting plasma cells or memory B cells for persistent protection [99, 128].

NK cells are the predominant cytotoxic T lymphocytes involved in innate immunity and mediate antitumour and antiviral responses. They display cytolytic activity that can be initiated through a variety of processes, including degranulation and death receptor ligation, and they produce several inflammatory cytokines, including TNF-α and IFN-γ [1, 79].

MCs and OCs are derived from CMPs. MCs mainly localize in the connective tissue, originate from granulocyte–macrophage progenitors (GMPs) and contribute to allergic responses and host protection against parasitic infection [7, 145]. Their cytoplasm contains large basophilic granules storing mediators of inflammation, such as histamine, heparin and serine proteases, and on their surface they express high-affinity immunoglobulin E (IgE) receptors (FCεRI), whose cross-linking by IgE causes their activation and the subsequent exocytosis of their granule content (degranulation) [70, 72].

OCs are giant, multinucleated, terminally differentiated cells that participate in bone homeostasis through their unique ability for bone resorption. They differentiate from the monocyte/macrophage lineage through two steps: they first proliferate in response to macrophage colony-stimulating factor (M-CSF) and therefore differentiate when the receptor activator of nuclear factor kappa-Β (RANK) on their surface is activated by its ligand RANKL, which is secreted by BM stromal cells and osteoblasts. RANKL/RANK signalling commits the precursors to differentiate into osteoclasts by activating the nuclear factor of activated T cells (NFATc1) to induce osteoclastogenic gene expression [25, 120].

### MiTF and HSCs homeostasis

At the top of the haematopoietic process, HSCs play a critical role in maintaining the appropriate number of terminally differentiated and fully functional blood cells during the entire lifespan of the organism. HSC attrition leads to BMF and anaemia and represents a driving force for MDS and leukaemia.

No specific alteration has been described in the HSC compartment in the *mi/mi* and *tg/tg* mice. However, a transient induction of MiTF has been recently identified during the awakening of quiescent HSCs in response to haematological stresses, such as blood loss or BM transplant. Following a stress that causes a need for new cells to replenish BM and/or circulating blood, the p38 MAPK pathway is activated in HSCs, with p38α promoting cell cycle entry and progression by stimulating purine metabolism [61]. MiTF was identified among several p38α targets and linked to the increased purine metabolism necessary to support the proliferative activity of awakened HSCs [61, 65, 73, 149]. Indeed, p38-activated MiTF binds to the promoter of the inosine monophosphate dehydrogenase 2 (IMPDH2) gene, which encodes a key rate-limiting enzyme of purine metabolism [61].

Moreover, MiTF enhances HSC homing and long-term engraftment downstream of a signalling cascade initiated by bone morphogenetic protein 4 (BMP4) in R-SMAD-dependent and -independent manner [23, 65]. BMP4 signalling leads to MiTF nuclear translocation, where it upregulates Integrin-α4 (ITGA4) expression, a transmembrane protein critical for HSC homing and retention in the BM [65, 125].

Another example of how MiTF deregulation can impact BM homeostasis was reported in a recent study we published on Fanconi anaemia (FA) [121]. FA, a rare genetic syndrome presenting developmental abnormalities of the skeleton, BM failure, leukaemia predisposition and genetic instability, is due to the loss-of-function of at least one of more than 22 genes that encode proteins constituting a major nuclear pathway involved in DNA repair and replication safeguards and rescue [11, 39, 115]. Interestingly, genome-wide and targeted analyses have shown that MiTF controls the expression of a set of genes involved in DNA replication and genomic stability in melanoma [165] and, in particular, acts as a critical regulator of the FANC pathway, which plays a key role in the proliferation and survival of melanoma, maintaining the high proliferative potential of melanoma cells and contributing to their high resistance to therapeutics [12]. Accordingly, in melanoma cells that constitutionally overexpress MiTF, its siRNA-mediated depletion leads to FANC protein downregulation and the entry of melanoma cells into senescence, at which point they accumulate chromosomal damage and mitotic abnormalities. In this context, MiTF-mediated FANC protein expression appears to be a requirement to cope with the high replication activity of tumour cells. Accordingly, the siRNA-mediated silencing of a FANC protein (FANCA or FANCD2) was sufficient to slow tumour growth, even when MiTF overexpression was maintained [12].

We recently extended the FA-MiTF connection demonstrating that MITF is overexpressed in cells from FA patients, suggesting a regulatory loop in which MiTF induces the FANC proteins that, in turn, downregulate MiTF expression/activity. More importantly, in contrast with the transient p38/MiTF activation observed during stress-induced haematopoiesis, we demonstrated that the p38/MITF axis is constitutionally active and associated with BMF in *Fanca*^−/−^ mice. Supporting the notion that the unscheduled and constitutional activation of the p38/MiTF axis has pathological consequences, p38 inhibition or siRNA-mediated depletion of MiTF was sufficient to rescue HSCs defects in the *Fanca*^−/−^ mice [121]. The previous observations shed light on two important physiologic aspects of the biology of both MiTF and FA. They show the key role of MiTF as a biological rheostat, which turns on and off drivers for optimal cell differentiation and functionality, and indicate that the deregulation of its "normal" activity/activation, more than its loss-of-function, leads to pathology. Timely regulated and transient expression of MiTF is important to replenish peripheral blood and BM, whereas its constitutive expression influences BM physiology, affecting HSCs self-renewal and quiescence. Therefore, the attrition of the HSCs pool observed in FA, which has been associated with the inability of cells to recover from DNA damage and p53/p21 axis overactivation, are clearly reinforced by the concomitant loss of the MiTF off switch.

### MiTF and lymphoid cells

**Fig. 3:**
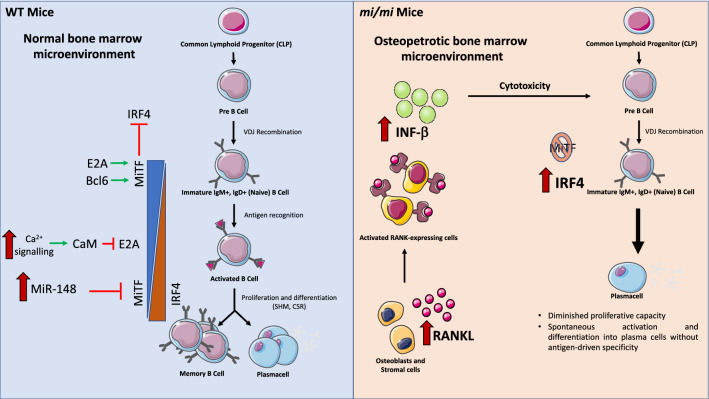
MiTF in B cells. In the normal bone marrow microenvironment, MiTF is highly expressed in naive B cells, where it acts as a repressor of IRF4. After B cell activation, MiTF is downregulated following the inhibition of the E2A factor and the upregulation of miR-148, leading to an increase in IRF4, which is essential for B cell terminal differentiation in antibody-secreting plasma cells. The decreased number of B cells and B cell precursors observed in *mi/mi* mice can be attributed to both direct and indirect effects of MiTF deficiency in these cells. The lack of MiTF in immature B cells causes an increase in IRF4 expression, leading to diminished proliferative capacity and spontaneous plasma cell differentiation in the absence of external stimuli. Moreover, mi/mi mice are characterized by an osteopetrotic microenvironment, which indirectly leads to the depletion of B cell precursors. In particular, osteoblasts and stromal cells secrete abnormal levels of RANKL to counteract osteoclast dysfunction, thereby activating RANK-expressing cells, which in turn secrete higher levels of IFN-β, which is known to induce cytotoxicity in B cell precursors

Lacking B cell precursors within the BM, *mi/mi* mice rely upon other lymphatic sites, such as the spleen, for B cell development and maturation. This phenotype was initially attributed to the osteopetrotic environment of the BM [140] and the presence of extracellular molecules, including RANKL, stromal-derived factor (SDF-1), B-cell lymphotactin chemokine (BLC) and interferon-β (IFN-β), which affect B cell behaviour [141] and [142]). However, in support of its direct involvement in B cell differentiation, MiTF is highly expressed in naive B cells to repress interferon regulatory factor 4 (IRF-4), a key factor for B cell activation and terminal differentiation in antibody-secreting plasma cells [85, 155]. Accordingly, defective MiTF activity results in spontaneous B cell activation and antibody and autoantibody secretion, while enforced MiTF expression suppresses the expression of IRF-4 and antibody secretion [85]. E2A [41] and Bcl6 [5], which maintain high levels of MiTF in naive B cells, are silenced downstream the antigen-mediated BCR activation through Ca^2+^ signalling-mediated calmodulin inhibition of E2A [41] and through the upregulation of miR-148a, which targets Bcl6 expression [131], leading MiTF downregulation and B cell terminal differentiation (Fig. 3).

Although NK cells differentiate normally in *mi/mi* mice, mi-Mitf impairs their cytotoxicity, sequestering key TFs or transcription regulators in the cytoplasm [46]. Thus, the expression of perforin, IL-12Rβ2, IL-18Rα, and the receptors for the proinflammatory cytokines IL-12 and IL-18, which mediate IFN-γ expression, is severely impaired in *mi/mi* NK cells [55, 63]. The relatively mild phenotype observed indicates that MiTF has a redundant function in NK cell physiology than can be performed by other proteins in its absence.

Consequently, MiTF is involved in the immune responses of organisms, acting as an important rheostat regulating immature B cell activation and terminal differentiation, as well as promoting in the optimal cytotoxicity of NK cells.

### MiTF and mast cells

Qi and collaborators identified the "pre-basophile and MC progenitors" (pre-BMPs), a subpopulation of the GMPs able to differentiate into basophiles or MCs [134]. A pre-BMP becomes a basophile when MiTF expression is silenced, while it will become an MC when MiTF expression is induced and maintained [145].

MiTF-A, MiTF-E, and MiTF-MC are the major isoforms expressed in MCs [150], which can also express MiTF-H and MiTF-M depending on physiological stimuli that modify both the promoter engagement and splicing [171]. MC proliferation is sustained by MiTF, which is induced by the stem cell factor (SCF)/c-kit signalling pathway, through the downregulation of miR-539 and miR-381 [78]. In turn, induced MiTF fosters c-kit expression. Accordingly, *Mitf*-mutated MCs respond poorly to SCF [26, 27, 172]. Other external stimuli such as IL-3, IL-4 and aggregated FCεRI proteins as well as activated PI3K pathway, p38α signalling and cytoplasmic adaptor protein SH3-binding protein 2 (3BP2) stabilize/increase MiTF protein synthesis or stability supporting MC differentiation [4, 50, 90, 114].

In MCs, MiTF is negatively regulated by its interaction with HINT [77] or PIAS3 [80]. Downstream IgE/FcεRI signalling activation, lysyl-tRNA synthetase (LysRS) is phosphorylated in a MAPK-dependent manner and translocated into the nucleus, where it produces the diadenosine oligophosphate Ap4A, which binds to HINT, liberating MiTF [77, 136, 184, 16]. The Mitf-PIAS3 interaction is lost as a consequence of MiTF phosphorylation in response to IL-6/IL-6R, SCF/Kit or IgE/FcεRI signalling activation [81, 158, 159].

Separated from its interactors, MITF is free to regulate several genes encoding key proteins involved in MC differentiation and activity, including (a) the granzyme B (GrB), which mediates the cytotoxic activity of MCs [53]; (b) the tryptophan hydroxylase (TPH), the rate-limiting enzyme for serotonin synthesis (Ito et al. [53]); (c) the histidine decarboxylase (Hdc), which regulates histamine synthesis [84], (d) the alpha-melanocyte-stimulating hormone (α-MSH) receptor (MC1R), which controls histamine release [2] and [3], and (e) the haematopoietic PGD2 synthase, which activates the cyclooxygenase pathway [106].

MiTF is also involved in the expression of several proteases stored in the secretory granules, including the endopeptidases MMCP-2, -4, -5, -6, -7, and -9 [32, 60, 101–103, 105, 109, 118, 119], the cathepsin G [60], and the transmembrane tryptase (TMT) [104]. MiTF regulates the expression of several adhesion molecules, including the integrin α4 subunit [66], which anchors the MCs to the extracellular matrix in BM and peripheral tissues [38], the plasminogen activator inhibitor-1 (PAI-1), involved in the regulation of extracellular matrix turnover through the inhibition of fibrinolysis [111], and the spermatogenic immunoglobulin superfamily adhesion molecule (SgIGSF) [56], which plays a role in MC migration [57, 107] and degranulation [58].

In quiescent MCs, MiTF interacts in the mitochondria with phosphorylated pyruvate dehydrogenase (PDH), a major regulator of the Krebs cycle that is essential for MC degranulation. In activated MCs, PDH is dephosphorylated and detached from MiTF to participate in degranulation. Thus, MiTF appears to be a negative regulator of PDH activity [151].

MiTF is also involved in the termination of MC-mediated responses. When the stimulus involved in the activation of MCs is resolved, the downregulation of MiTF contributes to the progression of apoptosis and the elimination of exhausted MCs [95, 173].

**Fig. 4:**
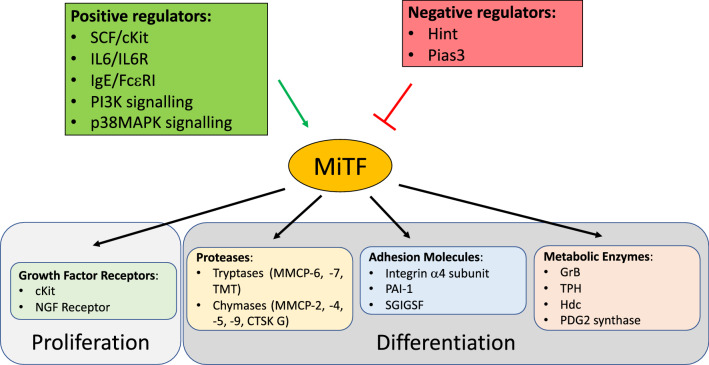
MiTF in mast cells. MiTF in mast cells is controlled by both positive and negative regulators: positive regulators can act either at the transcriptional level by increasing MiTF expression and at the post-transcriptional level by modulating MiTF activity and interactions with repressors, while negative regulators preferentially act at the protein level by binding to and inhibiting MiTF activity. Concerning its targets, MiTF controls the expression of proteins that are fundamental for both proliferation (i.e., growth factor receptors) and differentiation of mast cells. The latter include proteins stored in secretory granules, such as proteases, adhesion molecules and metabolic enzymes

In summary, MiTF is a key hub in MC physiology during their differentiation and activation, channelling several extracellular signals to target genes. Accordingly, MCs from *Mitf*-mutant mice appear immature, failing to express genes critical for MC functions [69, 160, 164], (Fig. 4).

### MiTF and osteoclasts

MiTF is a major determinant of the behaviour of osteoclasts and it is involved in bone remodelling and BM functionality. The MiTF-A isoform is steadily present in both progenitors and terminally differentiated OCs, whereas MiTF-E, undetectable in the progenitors, is strongly upregulated during osteoclastogenesis [86].

Osteopetrosis, a condition in which the bone hardens, becoming denser, indicating diminished or deficient osteoclast activity, is a major phenotypic characteristic of *mi/mi* mice [45]. Supporting the key role of MiTFs in the terminal differentiation of OCs, the number of their mononuclear precursors is normal in *mi/mi* mice [49, 94, 160, 169]. BM cell transplantation from normal donors rescues the osteopetrotic condition of the *mi/mi* mutant mice, demonstrating the haematopoietic origin of the OCs [178].

Interestingly, only the *mi/mi* mutation causes osteopetrosis; the *tg/tg* mice presented only minimal abnormalities during osteoclastogenesis (Steingrimsson et al. [161]). The previous difference in the osteopetrotic phenotype of the *mi/mi* and the *tg/tg* mice is due to two reasons: mi-Mitf sequesters partners involved in nuclear gene expression in the cytoplasm, and TFE3, expressed in the OCs, may serve as a backup for MiTF [30, 92, 93, 96, 180]. Accordingly, whereas osteoclasts appear normal in *tg/tg* and TFE3-null mice, the combined loss of the two genes leads to severe osteopetrosis (Hershey and Fisher 43; Steingrimsson et al. [162]). The MiTF/TFE3 redundancy establishes a critical role for the MiT family in osteoclastogenesis and provides an interpretation of why osteopetrosis in humans has not been related to MiTF genetic alterations.

Osteoclastogenesis depends on M-CSF and RANKL signalling. M-CSF promotes MiTF assembly with TFE3 in a MAPK/ERK signalling-dependent manner [181] or with PU.1 in an NADPH oxidase 2 (Nox2) and ERK signalling-dependent manner [113]. Thus, M-CSF promotes MiTF activity without increasing its protein level. In contrast, the combination of M-CSF and RANKL also increases the intracellular level of MiTF, mainly via the induction of the MiTF-E isoform. Indeed, RANKL induces the expression of the master osteoclast TF NFATc1, which supports MiTF-E expression [152] and in turn amplifies NFATc1-dependent gene transcription [87].

The interaction of RANKL, IL-1, TGFβ and BMPs with their receptors also induces MiTF by activating transforming growth factor β (TGFβ)-activated kinase 1 (TAK1) [74], which regulates the activity and/or expression of p38 MAPK, Smad1/5/8, NF-κB, MiTF, PU.1, c-Fos, and NFATc1 [92, 93, 133]. The intracellular level of MiTF can be elevated by heat shock factor 1 (HSF1), a transcriptional regulator of heat shock and cell stress responses, which enhances osteoclast differentiation [18, 19, 176] or by POH1 (pad one homologue), a deubiquitinating enzyme and component of the 26S proteasome that limits MiTF proteasomal degradation, increasing de facto its level [147, 148]. The transcriptional activity of MiTF is also fostered by p38 MAPK-dependent phosphorylation at its Ser-307, which allows the aggregation of a trimeric complex with the proto-oncogene FUS and the chromatin remodelling ATPase BRG1 [14].

Several factors inhibit MiTF activity in OCs. The Ikaros family protein Eos interacts with both MiTF and PU.1 to repress transcription at specific promoters through the recruitment of the corepressors Sin3A and CtBP [51]. In monocytic precursors, MiTF is excluded from the nucleus by its interaction with the chaperone-like adaptor 14-3-3 proteins and a Ser173 phosphorylated form of Cdc25C-associated kinase 1 (C-TAK) [13]. By inducing protein phosphatase 2A expression, RANK/RANKL signalling promotes pSer173 dephosphorylation, destroying the MiTF/C-TAK/14-3-3 complex and allowing MiTF nuclear translocation [148]. Several other factors bind and inhibit MiTF activity, including the MafB transcription factor [67], PIAS3 [47], the histone deacetylase 7 [127, 163], and inhibitors of differentiation/DNA binding (Ids) and helix-loop-helix (HLH) transcription factors [76]. Finally, at least two miRNAs inhibit MiTF expression in OCs: miR-155, which is induced by the TGFβ1/Smad4 pathway [189] or by interferon-β [187], and miR-340 [91], which interacts with the two binding sites on the 3′ untranslated region (UTR) of the MiTF mRNA, leading to its degradation (Goswami et al. [36]; Zhao et al. [190]).

MiTF regulates OC differentiation, fusion and activity via its interactions with several other TFs, including AP-1, PU.1, eomesodermin (EOMES or Tbr2) [15, 89, 116, 146], TFE3, TFEC, NFATc1, MEF2 or proteins involved in transcriptional regulation as the coactivator P300/CBP [181].

Through previous multiple interactions, MiTF regulates the expression of receptors and membrane-associated proteins involved in osteoclast fusion, such as the OC-associated receptor OSCAR (So et al. [157]); the subunit of the V-ATPase proton pump, V-ATPase d2 [29], the transmembrane 7 superfamily member 4 protein encoded by *DC-STAMP* [20], or the osteoactivin (OA) encoded by the *GPNMB* gene [139, 153]. Finally, MiTF participates in the expression of genes encoding proteins involved in the bone resorption activity of mature, terminally differentiated osteoclasts, such as the osteoclast metalloprotein tartrate-resistant acid phosphatase TRAP [88, 93], Partington et al. [126]); the cysteine protease Cathepsin K (CTSK), which plays an essential role in the degradation of protein components of bone matrix [97, 108, 124],and chloride channel 7 (Clcn7) and osteopetrosis-associated transmembrane 1 (Ostm1) proteins, which mutually localize at the membrane to regulate the acidity of the OC extracellular environment [98].

**Fig. 5:**
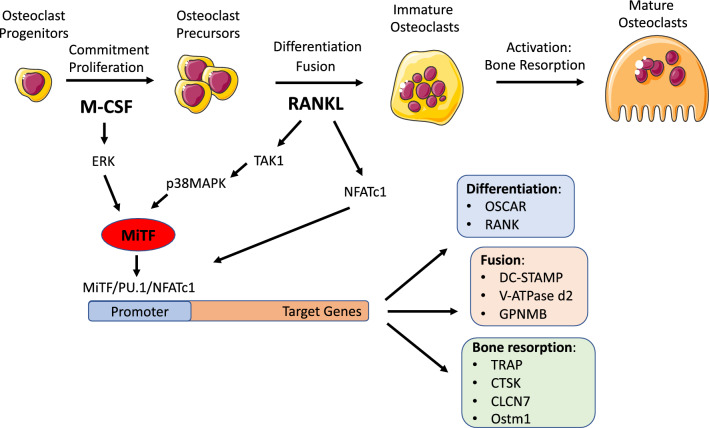
MiTF in osteoclast differentiation. MiTF is induced during osteoclastogenesis by both M-CSF, the cytokine responsible for the proliferation of osteoclast progenitors and their commitment towards an osteoclast fate, and RANKL, which induces the differentiation of osteoclasts through RANK signalling. M-CSF induces MiTF through the MAPK/ERK pathway, while RANKL signalling acts through the TAK1/p38 MAPK pathway. In parallel, RANKL also induces the expression of NFATc1, the other transcription factor essential for osteoclast differentiation. The MITF-PU.1 complex interacts with NFATc1 at osteoclast target gene promoters to initiate and maintain the expression of target genes. These genes encode markers of osteoclast differentiation, transmembrane proteins involved in the fusion of osteoclast precursors and enzymes involved in bone resorption

Thus, in OCs, MiTF represents the converging point for the activity of multiple signal pathways, which independently regulate each other or, in cooperative manner, regulate the MiTF expression level and/or activity, highlighting its major role in OC biology (Fig. 5).

## Conclusions

Since its discovery, MiTF has been shown to be a prominent key regulator of many aspects of melanocyte and melanoma biology. In this context, MiTF is unique among TFs for its ability to control a wide range of biological processes, such as cell proliferation, survival, differentiation, metabolism, invasion, senescence and DNA damage responses [35, 64]. Although still unclear, a master role for MiTF in some haematopoietic lineages cannot be ignored, notably in MCs and OCs, and for the homeostasis of the HSCs. On the previous basis, the expression of MiTF should be evaluated in other pathological situations in which BM fails, as well as in leukaemia, where its overexpression or loss-of-function may represent a driving force that allows the selection of clones bearing oncogenic mutations. For instance, subtle alterations in MiTF expression could participate in the emergence of leukemic clones in individuals with clonal haematopoiesis. The pharmacological modulation of the MiTF expression could be useful for care in several pathological setting, as bone marrow failure and bone abnormalities.
